# Proteome changes of porcine follicular fluid during follicle development

**DOI:** 10.1186/s40104-019-0400-3

**Published:** 2019-12-10

**Authors:** Victor M. Paes, Shengfa F. Liao, Jose R. Figueiredo, Scott T. Willard, Peter L. Ryan, Jean M. Feugang

**Affiliations:** 1 Department of Animal and Dairy Sciences, Mississippi State University, 4025 Wise Center, PO Box 9815, Starkville, Mississippi State MS 39762 USA; 2 Laboratory of Manipulation of Oocyte and Preantral follicles, State University of Ceará, Fortaleza, CE Brazil

**Keywords:** Follicular fluid, Folliculogenesis, Pig, Shotgun proteomic

## Abstract

**Background:**

Ovarian follicular fluid influences follicle and oocyte growth, but the fluctuation of its protein content during folliculogenesis has not been comprehensively analyzed. Here we used a shotgun approach and bioinformatics analyses to investigate and compare the proteomes of porcine follicular fluid (pFF) obtained from small (< 4 mm), medium (4–6 mm) and large (> 6–12 mm) follicles.

**Results:**

Follicular fluid samples containing highest estrogen levels were selected as non-atretic from small (SNA: 26.1 ± 15 ng/mL), medium (MNA: 162 ± 54 ng/mL), and large (LNA: 290 ± 37 ng/mL) follicles for proteomic analyses. We detected 1627, 1699, and 1756 proteins in SNA, MNA, and LNA samples, respectively. Nearly 60–63% of total proteins were specific to each sample, 11–13% were shared in pairwise comparisons, and 247 proteins were shared among all samples. Functional categorization indicated comparable gene ontology (GO) terms distribution per cellular component, molecular function, and biological process categories across samples; however, the ranking of highly significantly enriched GO terms per category revealed differences between samples. The patterns of protein-to-protein interactions varied throughout follicle development, and proteins such as serine protease inhibitor, clade E (SERPINE); plasminogen activator, urokinase (PLAU); and plasminogen activator, urokinase receptor (PLAUR) appeared stage-specific to SNA, MNA, and LNA, respectively. The “complement and coagulation cascades” was the common major pathway. Besides, properdin and fibulin-1 were abundant proteins that appeared absent in LNA samples.

**Conclusion:**

This study provides extensive and functional analyses of the pFF proteome changes during folliculogenesis and offers the potential for novel biomarker discovery in pFF for oocyte quality assessment.

## Background

Folliculogenesis and oogenesis in mammals occur in parallel within the ovary. These processes ensure the production of fully developmental competent oocytes that are capable of normal fertilization, followed by healthy embryonic development and birth of viable offspring [1]. Various intra- and extra-ovarian factors regulating these processes [2], allow for continuous follicle growth from preantral (~ 0.03 mm) to ovulatory (> 10 mm) stage together with its enclosed oocyte, from ~ 0.02 mm to 0.12 mm in diameter [3]. The transition from preantral to antral follicles is characterized by the formation of a cavity filled with a fluid also known as follicular fluid (FF), creating a microenvironment around the growing oocyte [4–7]. The FF derives from both ovarian follicle cells’ secretions and trans-exudate molecules (< 500 kDa) of the blood influencing follicle growth and oocyte maturation [8, 9]. The knowledge of the FF composition may provide unique insights into the processes regulating healthy follicle development, but its full characterization, especially its protein content, remains unfolded.

The dynamic composition of the FF during follicle growth has beneficial effects on the developmental competence of oocytes in larger follicles [10–13]. Numerous studies have found positive correlations between follicle size and oocyte developmental competence in various species [14–19]. For example, FF of medium-size follicles (5–8 mm) improves the maturation of oocytes collected from small-size follicles (2–5 mm) [20–22]. Indeed, FF is a vital and complex biofluid for female reproduction and constitutes an excellent source for discovering biomarkers of the follicle and/or oocyte growth [22–25]. In controlled experimental settings, numerous “reductionist” studies have reported the beneficial effects of various FF molecules (e.g., gonadotropins, steroids, and growth factors) on oocyte quality and resulting embryo development [26–32]. Despite the vast knowledge gained from these studies, they, unfortunately, do not extract the full benefit of the FF due to its complexity, developmental-stage variations, or molecular interactions. The identification of FF molecules contributing to these variations can help understand better the molecular mechanisms regulating follicle and oocyte growth.

Large-scale technologies, in opposition to the reductionist approaches, have been employed in human [33, 34], bovine [35, 36], horse [37], caprine [13], and porcine [38] FF to investigate their protein wealth under various ovarian physiological status. Most studies used gel-based proteomics, whereas few used gel-free or shotgun approaches to detect a higher number of proteins [39]. The current study uses the pig model to profile the proteome of FF harvested from different developmental stage follicles (small, < 4 mm; medium, 4–6 mm; and large, > 6–12 mm). Great number of proteins were generated for stronger predictions of molecular mechanisms or pathways underlying folliculogenesis and detection of potential biomarkers of follicle and oocyte growth.

## Materials and methods

### Ovarian follicle collection and follicular fluid aspiration

Approximately 240 ovaries were harvested from post-mortem sows (*n* = 120) at a local abattoir, in four independent replicates. Ovaries devoid of any active corpus luteum were kept on ice and transported to the laboratory within 2 h. Ovaries were washed with a 0.9% (*w*/*v*) NaCl solution supplemented with 1 μg/mL Penicillin/Streptomycin to eliminate the maximum of blood. Follicles were dissected from non-polycystic ovaries and classified as small (< 4 mm), medium (4–6 mm), and large (> 6–12 mm) diameters. Within the same ovary, follicular fluid (FF) of colorless and homogenous texture follicles were aspirated from individual large follicles and pools of at least four medium or four small follicles using appropriated needle fixed to a syringe. Fourteen to sixteen FF samples of individual large follicles and pools of medium or small follicles were constituted. All collected FF samples were centrifuged (1600×*g*; 5 min) at 4 °C to eliminate cells and debris. Supernatants were separately collected in clean tubes kept on ice, and aliquots (2× 25 μL) were taken for immediate quantifications of estradiol and total protein. The remaining sample was kept on ice until estradiol concentrations. This procedure was repeated for each independent ovary collections (*n* = 4 replicates).

### Estradiol (E_2_) and protein assays

For each ovary collection day, 25 μL of fresh FF sample was used to evaluate the E_2_ level, as described by the manufacturer (Estradiol ELISA kit; Calbiotech Inc., Spring Valley, CA, USA). Briefly, FF samples were thoroughly mixed with anti-E_2_ and E_2_-HRP conjugate in a 96-well plate and incubate at room temperature (22 °C) for 90 min. After removal of mixtures, all wells were washed and dried out on an absorbent paper. Wells were then refilled with the substrate to allow the reaction with HRP. After 20 min incubation at 22 °C, the reaction was stopped, and the absorbance was read at 450 nm with a microplate reader, within 15 min. A standard curve prepared in parallel was used to determine the E_2_ concentration in each sample. The sensitivity of the assay was 3.94 pg/mL and data (mean ± SD) are expressed as ng/mL. Intra-assay and inter-assay coefficients of variations were lower than 10% and 15%, respectively. Before sample assays, a preliminary validation test was conducted for a dose-dependent detection of a water-soluble E_2_, routinely used for assisted reproduction. After triplicate measurements of E_2_, remaining FF samples were pooled according to highest (non-atretic) and lowest (atretic) E_2_ levels within each follicle size category (first-level pooling according to E_2_ levels /replicate). Pooled samples were immediately stored at -80 °C until proteome analysis. This procedure was repeated for each independent ovary collections (*n* = 4).

For proteomic analysis, non-atretic (NA) FF samples with the highest estradiol level per ovary collection (~ 29 /follicle size) were thawed on ice and subsequently subjected to protein content analysis (Bradford reagent; Bio-Rad, Hercules, CA, USA). Thereafter, samples (*n* > 3) with comparable protein concentrations within each follicle size and ovary collection were pooled. Samples with comparable highest estradiol levels within each follicle size and ovary collection were selected for proteomic analyses. A total of four pooled samples (one per ovary collection) was used for each small (SNA), medium (MNA), and large (LNA) follicle category.

### Shotgun proteomics

The general procedure was performed as previously reported [39–41].

#### Sample cleanup method

Only three pools per follicle size category were used. Total FF protein (50 μg) of each follicle size was precipitated in 50% trichloroacetic acid (TCA) and subsequently subjected to trypsin digestion, as previously described [40]. Samples were adjusted to 2% acetonitrile and desalted using a peptide macrotrap (Michrom Bioresources, TR1/25108/52; Auburn, CA, USA). Each FF sample was loaded on a microtrap for wash (2% acetonitrile, 0.1% formic acid) to remove the digestion buffer, eluted (90% acetonitrile, 0.1% formic acid), and dried using vacuum centrifugation. Desalted samples were cleaned with a Strong Cation Exchange (SCX) macrotrap (Michrom TR1/25108/53) following manufacturer’s recommendation, to remove any detergents or other polymers that can interfere with MS/MS analysis. Each desalted and dried peptide pellet sample was resuspended in low salt buffer (5 mmol/L sodium phosphate, 25% acetonitrile, pH = 3 using formic acid), loaded on a SCX macrotrap (Michrom TR1/25108/53) for a second trap, and eluted with high salt buffer (5 mmol/L sodium phosphate, 25% acetonitrile, 0.25 mol/L potassium chloride, pH = 3 using formic acid). After cleaning, sample were dried (vacuum centrifugation), and resulted salt crystals and peptides were resuspended in 20 μL of 5% acetonitrile, 0.1% formic acid and transferred to a low retention autosampler vial for deconvolution via reverse phase, high-pressure liquid chromatography.

#### Nanospray LC-MS/MS method and protein identification

Each sample was loaded on a BioBasic C18 reversed phase column (Thermo 72105−100266) and flushed for 20 min with 5% acetonitrile (ACN), 0.1% formic acid to remove salts. Peptide separation was achieved using a Thermo Surveyor MS pump with a 655 min nano-HPLC method consisting of a gradient from 5% ACN to 50% ACN in 620 min, followed by a 20 min wash with 95% ACN and equilibration with 5% ACN for 15 min (all solvents contained 0.1% formic acid as a proton source). Ionization of peptides was achieved via nanospray ionization using a Thermo Finnigan nanospray source type I operated at 1.85 kV with 8 μm internal diameter silica tips (New Objective FS360–75-8-N-20-C12). High voltage was applied using a t-connector with a gold electrode in contact with the HPLC solvent. A Thermo LCQ DECA XP Plus ion trap mass spectrometer was used to collect data over the 655 min duration of each HPLC run. Precursor mass scans were performed using repetitive MS scans, each immediately followed by 3 MS/MS scans of the three most intense MS peaks. Dynamic exclusion was enabled with duration of 2 min and repeat count of two. Once a mass is measured twice, it is added to a list to be excluded from further analysis for a predetermined amount of time, which was 2 min. This allowed the MS to collect data on different masses, while in the meantime, the mass will have eluted from the column. Dynamic exclusion allows for a more efficient and deeper sample coverage. Mass spectra were searched against a protein database using the SEQUEST algorithm [42] in Bioworks 3.3 (Thermo Finnigan). The *Sus scrofa* NCBI RefSeq protein database was used for peptide spectral matching, and the Genome data from ENSEMBL was used for peptides that did not have corresponding proteins in the RefSeq database.

### Protein function and pathway identification

The distribution of total proteins detected across follicle sizes was performed with a Venn diagram (http://www.bioinformatics.psb.ugent.be/cgi-bin/liste/Venn/calculate_venn.htpl), followed by their annotation for biological, cellular localization, and molecular functions using the Agbase platform available with online website (https://agbase.arizona.edu/). Gene Ontology (GO) terms enrichment and pathway analyses were evaluated using SEA (Single Enrichment Analysis), and protein-to-protein interactions were assessed using STRING (http://www.string-db.org).

### Statistical analyses

Estradiol and protein concentrations were statistically analyzed with One-way ANOVA, followed by the Fisher’s LSD test. Search results for peptide matches were filtered using a decoy based, and proteins corresponding peptides with a probability of 0.05 or less were evaluated for further analyses. Bioinformatics analyses were performed using the default settings of each online software and protein association networks were obtained with highest confidence (interaction score > 0.9). The Benjamini-Hochberg False Discovery Rate (FDR) was set at 5% threshold.

## Results

### Follicular fluid estradiol and protein contents

The intrafollicular E_2_ levels of all analyzed samples varied from 0.12 to 49 ng/mL in small, 2 to 237 ng/mL in medium, and 6 to 500 ng/mL in large follicles. The E_2_ levels in constituted atretic samples were significantly lower than their nonatretic counterparts in small (1.53 ± 0.65 ng/mL vs. 19.54 ± 14.82 ng/mL), medium (11.52 ± 8.52 ng/mL vs. 150.28 ± 53.46 ng/mL), and large (32.25 ± 25.53 ng/mL vs. 311.03 ± 76.16 ng/mL) follicles (*P* < 0.05; ANOVA-1).

The average E_2_ levels in selected nonatretic samples for proteomic analyses were significantly (*P* < 0.05; ANOVA-1), and positively correlated with follicle sizes: 290 ± 37 ng/mL in large non-atretic (LNA), 162 ± 54 ng/mL in medium non-atretic (MNA), and 26 ± 15 ng/mL small non-atretic (SNA) follicles. Irrespective of the follicle health or size, the protein concentration of all porcine FF samples averaged 2.85 ± 0.05 μg/μL, without significant differences across follicle sizes (*P* > 0.05).

### Total protein identification and annotation

**Fig. 1:**
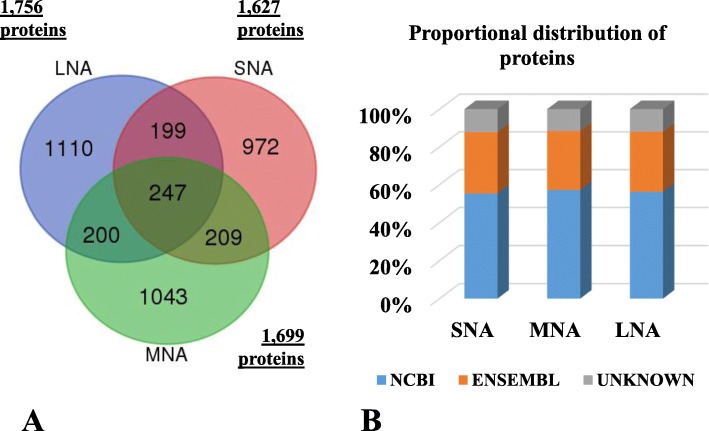
Total protein detection (**a**) and annotations (**b**). Venn Diagram of porcine follicular fluid was constructed with tools available at the Bioinformatics and Evolutionary Genomics, Ghent, Belgium

The total protein detected in each follicular fluid sample is summarized in Fig. 1a. A total of 1627, 1699, and 1756 unique proteins were detected in SNA, MNA, and LNA samples. The Venn diagram shows that approximately 60–63% of proteins were specific to SNA, MNA, and LNA, while 199, 200, and 209 proteins were shared between SNA-LNA, MNA-LNA, and SNA-MNA, respectively. Furthermore, 247 proteins were found in all follicle sizes. Approximately 55% and 31% of identified proteins were respectively annotated with NCBI and ENSEMBL, while 12% remained unknown (Fig. 1b). All proteome datasets are provided [see Additional file 1: Table S1].

### Abundant proteins

This analysis focused on the top 50 NCBI annotated proteins of all datasets [see Additional file 1: Table S1]. These proteins were considered highly abundant in datasets and corresponded to minimum detection probabilities of 5.94× 10^−28^, 2.25× 10^−27^, and 1.35× 10^−29^ in LNA, MNA, and SNA datasets, respectively. Most proteins (92%) were shared among datasets, the remaining were found in one or two different datasets and two others (properdin isoform X1 and fibulin-1 isoform X1) were completely absent in the LNA dataset. Specifically, properdin (XP_003135101) was detected at comparable level between SNA and MNA, while fibulin-1 (XP_003126003) was lower in MNA (vs. SNA).

### Bioinformatic analyses

#### Gene Ontology (GO) classification

The use of Agbase GO*Retriever* allowed the conversion of over 80% of protein datasets for functional categorization of identified GO terms in cellular component (CC), molecular function (MF), and biological process (BP) categories. Cell component (23–24%), cell (18–19%), intracellular (15–16%), membrane (12–13%), and cytoplasm (12–13%) GO terms in CC category (Table 1); binding (24–25%), protein binding (12%), molecular function (12%), catalytic activity (9%), hydrolase activity (7–8%) in MF category (Table 2); biological process (17%), cellular process (14%), metabolic process (11%), regulation of biological process (9%), macromolecule metabolic process (9%), and response to stimulus (7%) in BP category (Table 3). Regardless of the proteome datasets, the abundance of GO annotation/GO terms within each functional category did not show prominent differences.

**Table 1:** Functional classification of porcine follicular fluid proteomes during follicle development **-** Cellular component category

GO terms ~ GO names	Number of annotations per follicle developmental stage, %			Shared proteins
	SNA	MNA	LNA	*n*, %
GO:0005575 ~ Cellular component	435, 23.2	462, 23.6	479, 22.9	63, 21.0
GO:0005623 ~ Cell	345, 18.4	364, 18.6	379, 18.1	49, 16.3
GO:0005622 ~ Intracellular	286, 15.3	304, 15.5	336, 16.1	34, 11.3
GO:0016020 ~ Membrane	236, 12.6	242, 12.4	248, 11.8	29, 9.7
GO:0005737 ~ Cytoplasm	224, 11.9	233, 11.9	266, 12.7	24, 8.0
GO:0005634 ~ Nucleus	157, 8.4	155, 7.9	182, 8.7	21, 7.0
GO:0005576 ~ Extracellular region	76, 4.1	80, 4.1	82, 3.9	34, 11.3
GO:0005615 ~ Extracellular space	62, 3.3	62, 3.2	62, 3.0	35, 11.7
GO:0005694 ~ Chromosome	29, 1.5	30, 1.5	31, 1.5	3, 1.0
GO:0009986 ~ Cell surface	14, 0.7	13, 0.7	14, 0.7	4, 1.3
GO:0005578 ~ Extracellular matrix	11, 0.6	11, 0.6	14, 0.7	4, 1.3
Total annotations	1875, 100	1956, 100	2093, 100	300, 100

*SNA* Small non-atretic follicle (< 4 mm), *MNA* Medium non-atretic follicle (4–6 mm), *LNA* Large non-atretic follicle (> 6–12 mm)

**Table 2:** Functional classification of porcine follicular fluid proteomes during follicle development **–** Molecular function category

GO terms ~ GO names	Number of annotations per follicle developmental stage, %			Shared proteins
	SNA	MNA	LNA	*n*, %
GO:0005488 ~ Binding	365, 24.0	385, 24.7	408, 24.7	63, 24.0
GO:0005515 ~ Protein binding	178, 11.7	190, 12.2	203, 12.3	32, 12.2
GO:0003674 ~ Molecular function	175, 11.5	182, 11.7	200, 12.1	40, 15.3
GO:0003824 ~ Catalytic activity	143, 9.4	146, 9.4	152, 9.2	22, 8.4
GO:0016787 ~ Hydrolase activity	117, 7.7	114, 7.3	126, 7.6	20, 7.6
GO:0016740 ~ Transferase activity	105, 6.9	102, 6.5	99, 6.0	13, 5.0
GO:0003676 ~ Nucleic acid binding	103, 6.8	105, 6.7	107, 6.5	12, 4.6
GO:0005215 ~ Transport activity	55, 3.6	43, 2.8	42, 2.5	13, 5.0
GO:0016301 ~ Kinase activity	50, 3.3	45, 2.9	38, 2.3	6, 2.3
GO:0015075 ~ Ion transmembrane transporter activity	43, 2.8	31, 2.0	31, 1.9	4, 1.5
GO:0030234 ~ Enzyme regulator activity	41, 2.7	42, 2.7	46, 2.8	23, 8.8
GO:0016491 ~ Oxidoreductase activity	29, 1.9	29, 1.9	33, 2.0	3, 1.1
GO:0005198 ~ Structural molecular activity	27, 1.8	36, 2.3	43, 2.6	7, 2.7
GO:0004872 ~ Signaling receptor activity	24, 1.6	45, 2.9	42, 2.5	2, 0.8
GO:0015267 ~ Channel activity	23, 1.5	18, 1.2	15, 0.9	3, 1.1
GO:0003774 ~ Motor activity	10, 0.7	11, 0.7	19, 1.1	1, 0.4
GO:0016829 ~ Lyase activity	10, 0.7	7, 0.4	10, 0.6	1, 0.4
GO:0016209 ~ Antioxidant activity	7, 0.5	6, 0.4	5, 0.3	1, 0.4
GO:0016853 ~ Isomerase activity	7, 0.5	5, 0.3	6, 0.4	–
GO:0016874 ~ Ligase activity	7, 0.5	8, 0.5	13, 0.8	1, 0.4
GO:0004386 ~ Helicase activity	2, 0.1	6, 0.4	5, 0.3	–
GO:0009055 ~ Electron transfer activity	1, 0.1	1, 0.1	6, 0.4	–
GO:0045182 ~ Translation regulator activity	1, 0.1	1, 0.1	2, 0.1	–
Total annotation	1523, 100	1559, 100	1654, 100	262, 100

*SNA* Small non-atretic follicle (< 4 mm), *MNA* Medium non-atretic follicle (4–6 mm), *LNA* Large non-atretic follicle (> 6–12 mm)

**Table 3:** Functional classification of porcine follicular fluid proteomes during follicle development **–** Biological process category

GO terms ~ GO names	Number of annotations per follicle developmental stage, %			Shared proteins
	SNA	MNA	LNA	*n*, %
GO:0008150 ~ Biological process	582, 17.0	610, 17.2	639, 17.2	110, 17.7
GO:0009987 ~ Cellular process	484, 14.2	503, 14.2	537, 14.4	83, 13.3
GO:0008152 ~ Metabolic process	385, 11.3	397, 11.2	416, 11.2	78, 12.5
GO:0050789 ~ Regulation of biological process	313, 9.2	321, 9.0	335, 9.0	63, 10.1
GO:0043170 ~ Macromolecule metabolic process	304, 8.9	309, 8.7	329, 8.8	69, 11.1
GO:0050896 ~ Response to stimulus	241, 7.0	248, 7.0	244, 6.6	54, 8.7
GO:0007154 ~ Cell communication	150, 4.4	171, 4.8	173, 4.6	27, 4.3
GO:0009058 ~ Biosynthetic process	148, 4.3	152, 4.3	177, 4.8	21, 3.4
GO:0006139 ~ Nucleobase-containing compound metabolic process	141, 4.1	145, 4.1	171, 4.6	17, 2.7
GO:0006810 ~ Transport	120, 3.5	116, 3.3	120, 3.2	20, 3.2
GO:0032501 ~ Multicellular organismal process	118, 3.5	125, 3.5	126, 3.4	24, 3.9
GO:0007275 ~ Multicellular organismal development	100, 2.9	102, 2.9	109, 2.9	10, 1.6
GO:0030154 ~ Cell differentiation	81, 2.4	85, 2.4	81, 2.2	10, 1.6
GO:0009056 ~ Catabolic process	65, 1.9	65, 1.8	71, 1.9	9, 1.4
GO:0006928 ~ Movement of cell or subcellular component	49, 1.4	53, 1.5	48, 1.3	6, 1.0
GO:0051704 ~ Multi-organism process	42, 1.2	48, 1.4	57, 1.5	9, 1.4
GO:0008219 ~ Cell death	38, 1.1	43, 1.2	37, 1.0	6, 1.0
GO:0046903 ~ Secretion	20, 0.6	28, 0.8	22, 0.6	3, 0.5
GO:0043062 ~ Extracellular structure organization	15, 0.4	10, 0.3	10, 0.3	4, 0.6
GO:0006520 ~ Cellular amino acid metabolic process	12, 0.4	7, 0.2	12, 0.3	–
GO:0007610 ~ Behavior	7, 0.2	12, 0.3	6, 0.2	–
GO:0006944 ~ Membrane fusion	4, 0.1	1, 0.0	4, 0.1	–
Total annotation	3419, 100	3551, 100	3724, 100	623, 100

*SNA* Small non-atretic follicle (< 4 mm), *MNA* Medium non-atretic follicle (4–6 mm), *LNA* Large non-atretic follicle (> 6–12 mm)

#### Gene Ontology (GO) and enrichment analyses

**Fig. 2:**
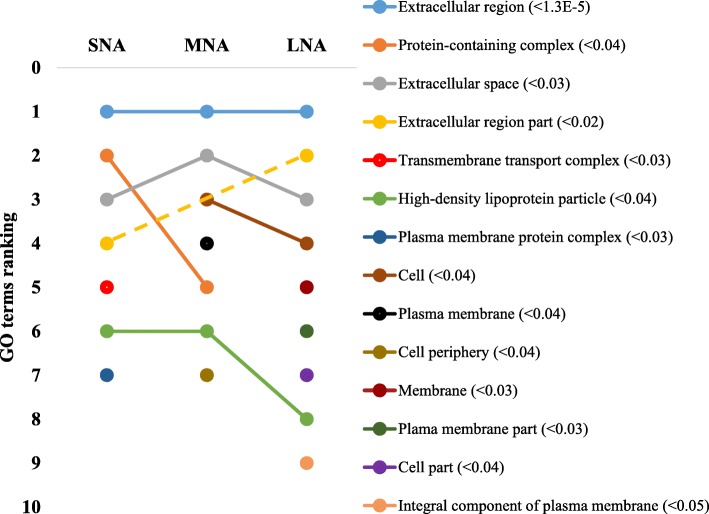
Dynamic ranking of the top enriched GO terms in the component cellular category. Significant enrichments were declared at *P* < 0.05. GO terms were ranked from 1 to 10 according to their degree of significance, based upon the false discovery rates or FDR values (in parentheses). Dotted line represents GO terms that did not show significant enrichments in MNA samples

**Fig. 3:**
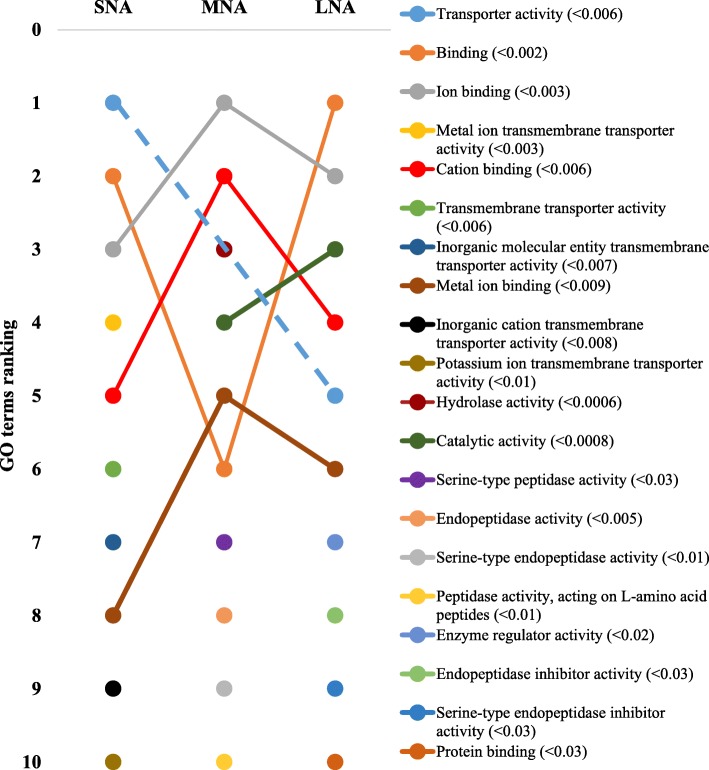
Dynamic ranking of the top enriched GO terms in the molecular function category. Significant enrichments were declared at *P* < 0.05. GO terms were ranked from 1 to 10 according to their degree of significance, based upon the false discovery rates or FDR values (in parentheses). Dotted line represents GO terms that did not show significant enrichments in MNA samples

**Fig. 4:**
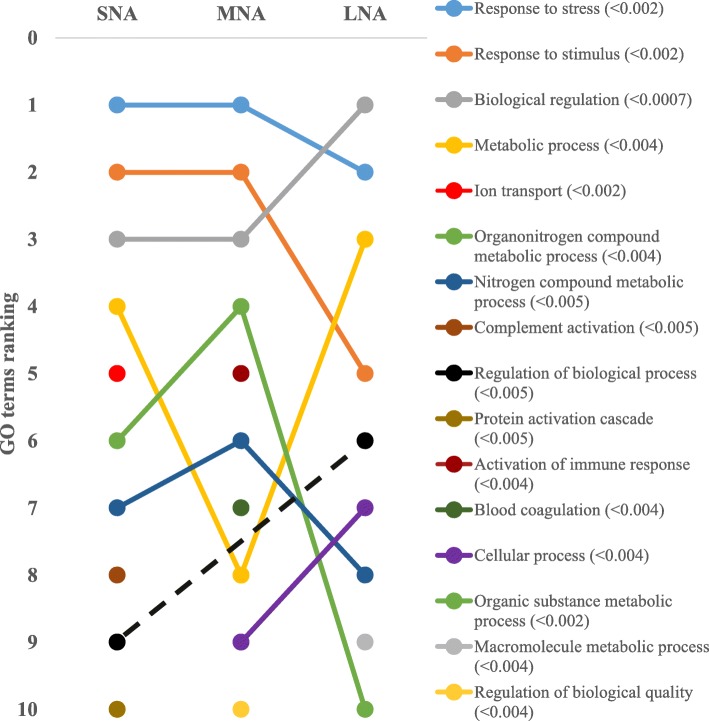
Dynamic ranking of the top enriched GO terms in the biological process category. Significant enrichments were declared at *P* < 0.05. GO terms were ranked from 1 to 10 according to their degree of significance, based upon the false discovery rates or FDR values (in parentheses). Dotted line represents GO terms that did not show significant enrichments in MNA samples

The STRING software allowed respective conversions of 1054, 1089, 1139 proteins in SNA, MNA, and LNA datasets, corresponding to ~ 65% of total proteins (see Additional file 2: Table S2). The highly significantly enriched GO terms in CC, MF, and BP were ranked according to significance levels in Figs. 2, 3, and 4, respectively. Dynamic ranking (increase, stable, or decrease) of GO terms were observed across SNA, MNA, and LNA.

In Fig. 2, proteins associated with “extracellular region” GO term were the highest and significantly enriched (FDR < 1.3 × 10^−5^) in all follicle category. Meanwhile, fluctuation and decrease significances in protein enriched were observed within the “protein-containing complex”, “extracellular space”, “cell”, and “high density lipoprotein particle” GO terms throughout follicle growth. In contrast, the significant enrichment of proteins in other GO terms (i.e., “extracellular region part”, membrane”, “plasma membrane part”), made them pro-imminent GO terms in LNA samples. Unlike in CC, the GO terms’ patterns in MF showed high ranking fluctuations (Fig. 3). Terms such as “transporter activity”, “binding”, “cation binding”, and metal ion binding” highly fluctuated across samples, to become the most enriched GO terms in LNA, together with “catalytic activity”. The patterns in the BP category (Fig. 4) revealed similar fluctuations, mainly observed between MNA and LNA samples with decreased ranking of “response to stress”, “response to stimulus”, “organonitrogen compound metabolic process”, and “nitrogen compound metabolic process”. “Biological regulation”, metabolic process”, regulation of biological process”, “cellular process” GO terms were highly ranked in LNA, while “ion transport”, “complement activation”, and “protein activation cascade” were not significantly ranked as compared to LNA. Interestingly, proteins such as serine protease inhibitor, clade E (SERPINE); plasminogen activator, urokinase (PLAU); plasminogen activator, urokinase receptor (PLAUR) were detected only in SNA, MNA and LNA, respectively.

#### Protein-to-protein interactions (PPI) and pathway analyses

**Fig. 5:**
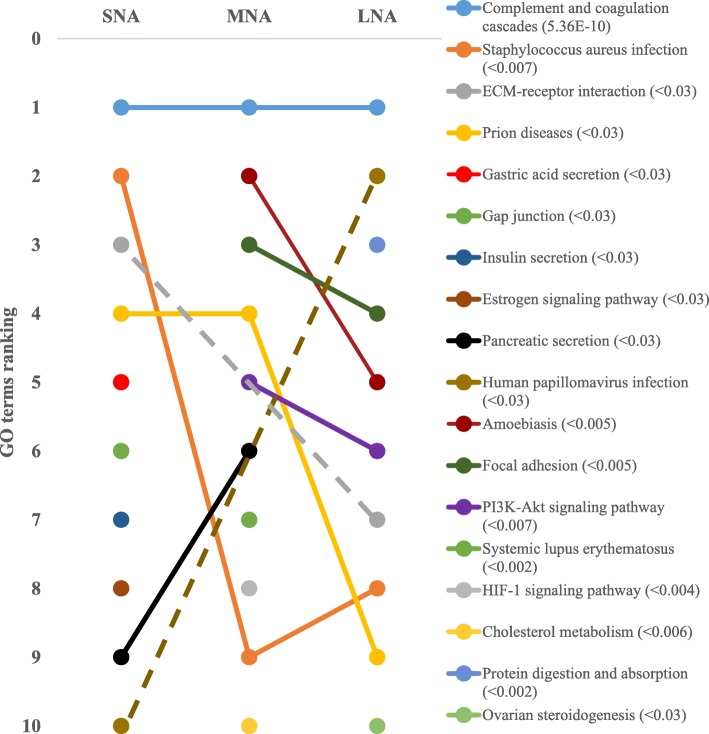
Dynamic ranking of the top enriched GO terms pathway. Significant enrichments were declared at *P* < 0.05 and false discovery rates or FDR values are shown in parentheses. Dotted lines represent GO terms that did not show significant enrichments in MNA samples

Data were generated through significantly enrichment (*P* < 10^−16^) and highest detection confidence (0.9). Kmeans clustering networks showed variable patterns of PPI in SNA, MNA, and LNA, and “complement and coagulation cascades” appeared as the major KEGG pathway in all samples (Fig. 5; FDR < 5.36 × 10^−10^). Other pathways such as “focal adhesion”, “protein digestion and adsorption”, “PI3K-Akt signaling pathway”, “ECM-receptor interaction”, and ovarian steroidogenesis” were highly ranked in LNA samples.

**Fig. 6:**
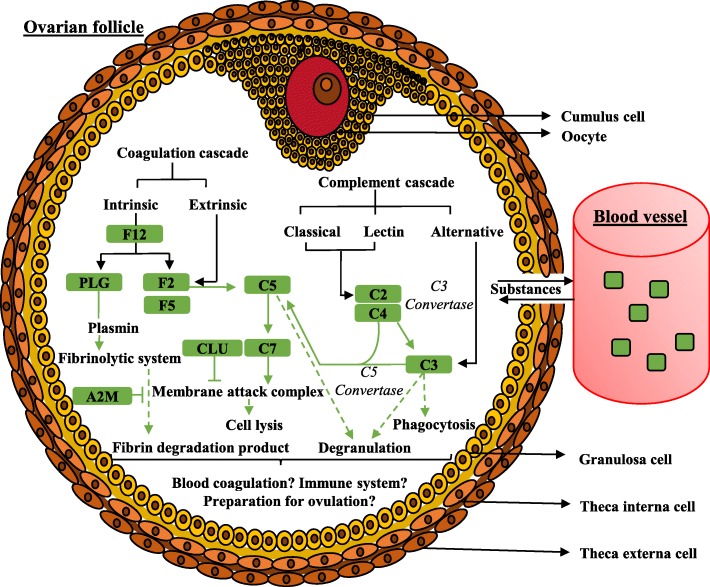
Schematic representation of the “complement and coagulation cascades” pathway within the ovarian follicle. Pathways were generated through commonly shared proteins (or core-proteins), highlighted green, found across all datasets

The Fig. 6 schematizes the “complement and coagulation cascades” pathway with contributing proteins of our datasets, such as F12 (Coagulation factor XII); F2 (Thrombin); C5 (Complement 5a anaphylatoxin); C2 (Complement component 2); C3 (Complement 3); C7 (Complement component 7); CLU (Clusterin); A2M (Alpha-2-macroglobulin), F5 (Coagulation factor V); C4 (Uncharacterized protein; complement C4); PLG (Plasminogen); SERPIN (Serine peptidase inhibitor) that were found in all follicle categories.

## Discussion

Ovarian follicular fluid is rich in various biocompounds (i.e., fatty acids, metabolites, proteins, steroids) that diversely and time-mannerly regulate oogenesis and folliculogenesis [26–32]. Previous studies have demonstrated differential developmental competence of oocytes [17–19] derived from small (SNA: < 4 mm), medium (MNA: 4–7 mm) and large (LNA: > 6–12 mm) follicles [20, 21, 38], while others revealed the dynamic content of the follicular fluid playing critical roles during folliculogenesis [20, 21]. The present study characterizes the proteomes of specific developmental stage ovarian follicular fluids in pigs. It provides sets of predicted protein functions and networks that will contribute to the identification of key regulators of follicle and oocyte growth and potential biomarkers for oocyte quality assessment in assisted reproduction.

In the present study, colorless follicles with homogenous texture were selected and classified as small, medium, and large. Because of their reduced volumes, follicular fluids (FF) of different small or medium follicles of the same ovary were mixed during collection. A further reduction of individual various among ovaries and pigs was achieved by pooling FF of comparable higher or lower estradiol levels within the same follicle size; high estradiol levels in FF have been reported as features of healthy and non-atretic follicles [43, 44]. The follicle size-dependent increase of estradiol was in agreement with previous studies primate human [45] and non-human [46], rat [47] and livestock [48, 49], reporting association between estradiol synthesis and follicular cell viability. In contrast, intrafollicular protein concentrations were comparable regardless of their origins, and were consistent with previous findings in pigs [50], cows [51], buffaloes [52], and goats [13].

The benefits of proteomic tools in the study of ovarian follicular fluids have been highlighted in various species, despite its known limitations (e.g., dynamic range of samples [53]. In the present work, proteome analyses revealed follicle size-dependent protein contents of follicular fluids with 1627, 1699 and 1756 proteins in SNA, MNA and LNA, respectively. This increase total protein numbers contrasted with the unchanged protein concentrations across samples, but therefore confirmed the increasing FF volume throughout follicle growth. A similar increase pattern of total protein numbers across follicle sizes has been reported in bovine FF [35]. It is speculated that protein increased numbers may result from higher 1) secretions of granulosa and thecal cells and 2) permeability of the ovarian-blood-follicle barrier allowing more protein flux into the FF to support follicle growth and oocyte maturation.

Subsets of proteins were specifics to SNA (972), MNA (1043), and LNA (1110) samples, while other 200 overlapping proteins were qualified as “transitional proteins”, between SNA-MNA, MNA-LNA, or SNA-LNA. These proteins may likely play critical roles during folliculogenesis progression (See Additional file 1: Table S1). Meanwhile, 247 proteins qualified as “core or housekeeping proteins” of folliculogenesis/oogenesis were common to all follicle sizes. A similar protein distribution has been reported in other species, including human [54], buffalo [12], goat [13], and horse [37, 55].

Focusing on the 50 most abundant proteins with NCBI annotations, 92% (46) of proteins was found across all follicle developmental stages. As expected, proteins such as serum albumin, fibrinogen beta chain, serotransferrin, ceruloplasmin, immunoglobulin G, serpin A3–8, alpha-2-HS-glycoprotein, and inhibitor of carbonic anhydrase were among the highly abundant proteins across samples. Within the abundant protein lists, fibulin-1 and (complement factor) properdin were not detected in the LNA samples. Fibulin-1 is a calcium-binding glycoprotein found in the blood that participates to the extracellular matrix organization and plays a role in thrombosis leading to blood clot [56]. In the present study, the absence of fibulin-1 in the LNA following its lower detection in MNA are indicative of the follicle’s preparation for the ovulation process that could be impeded by blood clotting. Furthermore, properdin is a member of the complement family found in the blood plasma and stabilizing the alternative pathway of the complement system (C3 and C5 convertases) by extending the half-life of the C3 and C5 converting enzymes [57]. The expression of properdin seems associated with early apoptotic cells and its absence in LNA and comparable detection levels in SNA and MNA samples coincide with the incidence of apoptosis during folliculogenesis.

The current study provides the largest proteome datasets of follicular fluids harvested from different developmental stage healthy porcine follicles. Given the current limitation of the pig genomic database, only 56% and 32% of total proteins were respectively annotated with NCBI and ENSEMBL databases. Interestingly, the combination of both database annotations led to higher proportion (88%) of protein annotations for fundamental predictions of protein functions through gene ontologies.

Gene ontology (GO) analyses revealed higher proportions (> 41%) of both “cellular component” and “cell” GO terms in cellular component category, while over 35% of general “binding” and “protein binding” GO terms in molecular function category and 42% of “biological process”, “cellular process”, and “metabolic process” GO terms in biological process category were the most prominent in all generated proteome datasets. These distributions were consistent with previous reports in bovine [35] and caprine FF [13]. The high proportions (> 15%) of intracellular proteins found in all proteome datasets were in agreement with a report in buffalo [12], and may reflect the perpetual remodeling (apoptosis and/or mitogenic turnover rate) of granulosa cells to support osmotic force of the growing liquid volume in the antrum cavity [58, 59]. In all proteome datasets, proteins associated with “extracellular region” were the highly enriched, while the FDR-based ranking of GO terms revealed the high significance of “high-density lipoprotein particle” GO term correlating with high level of lipid in follicular fluid [5]. Also, high enrichments of “binding”, “ion binding”, “metal ion binding”, and “cation binding” GO terms coincide with reported high levels of albumin and immunoglobulin in follicular fluids [12, 13, 37]. The FDR-based ranking revealing great fluctuations of significantly enriched GO terms (per functional category) across proteome datasets indicates likely changes of protein functions throughout folliculogenesis that can affect female fertility. Further investigations of critically enriched GO terms could permit the identification of potential protein candidates for follicle growth and/or oocyte maturation.

In the current study, numerous proteins belonged to several KEGG pathways. Among the most interesting, pathways such as “*Staphylococcus aureus* infection”, “prion disease”, “gastric acid secretion”, “insulin secretion”, “human papillomavirus infection”, “systemic lupus erythematosus”, and “amoebiasis” exhibited higher significances in SNA and MNA, while LNA samples were characterized by pathways such as “protein digestion and absorption”, “focal adhesion”, and “PI3K-Akt signaling pathway”. In agreement with previous reports in women [60], goats [13] and mares [55], the “complement and coagulation cascades” was the highly significant pathway across all samples. Both cascades shared protein inhibitors and activators of serine endopeptidase that are associated with inflammatory response [61], a required process for ovulation [62]. Numerous proteins that contribute to blood coagulation and fibrin degradation through plasminogen (PLG) or proteolysis by thrombin activation [61] were detected. For example, alpha-2-macroglobulin (A2M), a protease inhibitor that binds small or large proteinases such as the plasmin that regulates proteolytic activity [63], influences cumulus cells expansion by inhibiting zinc-dependent metalloproteases [64]. In this way, the excess of A2M lead a reduction in porcine cumulus expansion [64, 65]. Clusterin (CLU), another protease inhibitor, is an important regulator of the complement [66], preventing apoptosis of follicular cells [67].

The current study reveals the presence of serine protease inhibitor clade E (SERPINE) and plasminogen activator urokinase (PLAU) in SNA and MNA follicular fluids, respectively. Both proteins have shown potential roles on cumulus cells expansion in a previous study [68]. On the other hand, the detection of PLAU receptors (PLAUR) in large follicles suggests a possible role of the PLAU/PLAUR complex at the later stage of folliculogenesis to favor oocyte maturation. We speculate that the establishment of this complex allows cell-surface plasminogen activation through its conversion into plasmin inducing localized degradation of the extracellular matrix, which in turn influence the expansion of cumulus cells surrounding the maturing oocyte and prepare the ovarian tissues for ovulation.

## Conclusion

Our study provides the proteome profiles of pFF during follicle growth for comparative analyses. Functional analyses of proteome datasets revealed protein clusters and networks that constitute the “core signature” of folliculogenesis, from which any deviations may serve for developmental biomarkers search. Hierarchical GO term arrangements were indicative of (qualitative and/or quantitative) protein variations during follicle growth. Several detected proteins and functions were already known, and the current study highlights follicle developmental-stage specific proteins that regulates oocyte maturation in small (SERPINE), medium (PLAU), and large (PLAUR) follicle sizes. Meantime, properdin and fibulin-1 were found as proteins of interest for follicle growth. The proposed datasets provide a useful basis for future studies to better comprehend ovarian folliculogenesis.

## Supplementary information

**Additional file 1:** **Table S1.** Proteome datasets.

**Additional file 2:** **Table S2.** Functional analyses.

## Data Availability

All data generated or analyzed during this study are included in this published article [and its supplementary information files].

## Abbreviations

A2M: Alpha-2-macroglobulin
BP: Biological process
C2: Complement component 2
C3: Complement 3
C4: Uncharacterized protein, complement C4
C5: Complement 5a anaphylatoxin
C7: Complement component 7
CC: Cellular component
CLU: Clusterin
E_2_: Estradiol
F12: Coagulation factor XII
F2: Thrombin
F5: Coagulation factor V
FF: Follicular fluid
GO: Gene ontology
LNA: Large non-atretic
MF: Molecular function
MNA: Medium non-atretic
PLAU: Plasminogen activator, urokinase
PLAUR: Plasminogen activator, urokinase receptor
PLG: Plasminogen
PPI: Protein-to-protein interactions
SERPIN: Serine peptidase inhibitor
SERPINE: Serine protease inhibitor, clade E
SNA: Small non-atretic
